# Supplementary material for: Predicting eye movement patterns from fMRI responses to natural scenes
Source: Nat Commun. 2018 Dec 4;9:5159. doi: 10.1038/s41467-018-07471-9 (PMC6279768; doi:10.1038/s41467-018-07471-9)
Supplement: Supplementary file 1 — Supplementary Information [file 41467_2018_7471_MOESM1_ESM.pdf]

Supplementary Information for
O'Connell and Chun, Predicting eye movement patterns from fMRI responses to natural scenes

SUPPLEMENTARY METHODS

Training spatial attention models with eye movements. In the spatial attention modeling literature, most models are trained by fitting predicted spatial priority maps to actual eye movement patterns. Additionally, many models restrict spatial predictions using empirically-derived baseline fixation distributions. While both of these steps improve prediction performance, both are dependent on signals derived from eye movement behavior. We excluded any analytic step in our model-based pipeline that is dependent on eye movement data to ensure that accurate predictions from model-based reconstructions were fully zero-shot. For the spatial attention model, we achieved this by averaging across CNN channels and preserving the channel weightings learned during goal-directed training for a given type of visual recognition, rather than learning to re-weight channels by explicitly linking CNN activity to eye movements. For the decoding models, we achieved this by not conditioning or modifying the reconstructions using any signal derived from eye movement data.

Here, we present two analyses more consistent with the mainstream spatial attention modeling literature. The first analysis shows that explicitly re-weighting CNN activity to predict eye movements improves prediction performance for decoding models derived from face-identification and random CNNs, but not scene- and object-categorization CNNs. The second analysis shows that model-based fMRI prediction performance can be improved using an empirically-defined baseline fixation distribution to correct for center-bias.

Re-weighting CNN activity to explicitly predict fixation patterns. Here, we aim to re-weight CNN activity to improve prediction performance in the spatial attention models and model-based reconstructions. Such an approach tests whether features from a given CNN type map onto spatial representations in the brain that predict eye movements if an additional learning step explicitly linking CNN activity to eye movements is included.

To learn weights across CNN channels that improve eye movement prediction, we predicted fixation map values from CNN unit activity drawn from the five pooling layers (1,472 channels total) using support-vector regression with a ridge penalty on data from the MIT Eye Movement Dataset [1]. Activity maps for each CNN channel were linearly interpolated to the spatial resolution of pool1 (112 x 112 px) and all re-sized units within a layer were normalized to have zero-mean and unit standard deviation. For each of the 1000 training images, this produced a [1472 x 112 x 112] matrix of CNN activity. Fixation maps were calculated for each of the training images at the group-level (n = 15) and smoothed using a 2D Gaussian kernel (SD = 20 px, matched to the cross-validated smoothing kernel from our validation datasets). For each training image, we randomly sampled 100 image locations to build the data matrix for the regression. For a given sampled location, the fixation map value becomes a new Y and the CNN activity values across all 1472 channels become a new row of X's, leading to a final Y vector of [100,000 x 1] and an X matrix of [100,000 x 1472]. The regression outputs a [1 1472] vector of

beta weights that can be multiplied by a [1472 12544] matrix of CNN activity for a given image to re-weight the activity to better predict eye movements.

Using the learned weighting to calculate computational spatial priority maps from CNN activity improved prediction performance for all CNN types (**Supplementary Fig. 1a**). Modest improvements were seen for the scene- and object-categorization CNNs, and markedly greater improvements were seen for the face-identification and random CNNs.

Next, we show results for predicting eye movements using model-based reconstructions for each CNN type that average across channels or computed a weighted sum across channels. *NSS* scores can be seen for all analysis types and ROIs in (**Supplementary Fig. 1b & 1d**) and *NSS* difference scores (average model – weighted model) can be seen in (**Supplementary Fig. 1b & 1d**). Significance markers for the difference scores in **Supplementary Fig. 1** represent the main effect for model type (average vs weighted) in a 2-way repeated-measures ANOVA with model type and ROI as factors. We find that performance is equivalent for the average and weighted approaches for decoding models using scene CNNs (**Supplementary Fig. 1c & 1e, first column**) for within-individual and internal validations. For external validation, the average model outperformed the weighted model for base reconstructions and equivalent performance was seen for smoothed and center-bias corrected reconstructions. For base and smoothed/center-bias corrected reconstructions from object CNNs (**Supplementary Fig. 1c & 1e, second column**), performance was equivalent for within-individual validation, and the average model outperformed the weighted model for internal and external validation. For base and smoothed/center-bias corrected reconstructions from face and random CNNs (**Supplementary Fig. 1c & 1e, third and fourth columns**), performance was equivalent for within individual validation, but the weighted model outperformed the average model for internal and external validation.

Overall, these results show that features optimized for scene and object categorization best generalize off-the-shelf to characterize spatial representations in visual brain regions that predict eye movements. For scene and object categorization CNNs, re-weighting was not necessary to get respectable computational and brain-based predictions; the averaging model using the relative weighting of channels set through learning to complete the visual categorization task already well captures spatial contingencies in scenes that are consistent with eye movement patterns, both behaviorally and in the brain. The relative weighting amongst channels for the face and random CNNs does not capture spatial information relevant to predicting eye movements by default; an additional explicit learning step is necessary to achieve performance comparable to the scene and object CNNs off-the-shelf.

Empirical center-bias correction. To correct for center-bias empirically, the center-model was defined as a baseline fixation distribution across all images except the target image in a separate set of participants. Calculation of these baseline distributions was cross-validated across data sets (internal and external validation). For example, the empirical baseline for Image A in the within-individual or internal validation analyses was defined as the average fixation density map for all other images and all participants in the external validation dataset. The empirical baseline for the same image in the external validation analyses was the average fixation density map for all other images and all participants in the internal validation dataset. Each empirical baseline was re-

scaled from 0 to 1. To correct for center-bias in the reconstructions using these empirically-derived baselines, we pointwise multiply the baselines with a spatial priority map reconstruction after the reconstruction has been smoothed with a 2D Gaussian kernel. The procedure is the same as for the Gaussian center-bias correction used in our primary analyses.

Empirical correction for center-bias improved reconstruction prediction performance across the within-individual, internal validation, and external validation analyses (**Supplementary Fig. 2**). As for the model-based reconstructions presented in the manuscript, empirically center-bias corrected reconstructions from V1, V2, V3, and hV4 significantly predicted eye movement patterns in the within-individual, internal validation, and external validation analyses (all $P<0.001$). Example reconstructions can be seen in (**Supplementary Fig. 3**).

SUPPLEMENTARY FIGURES

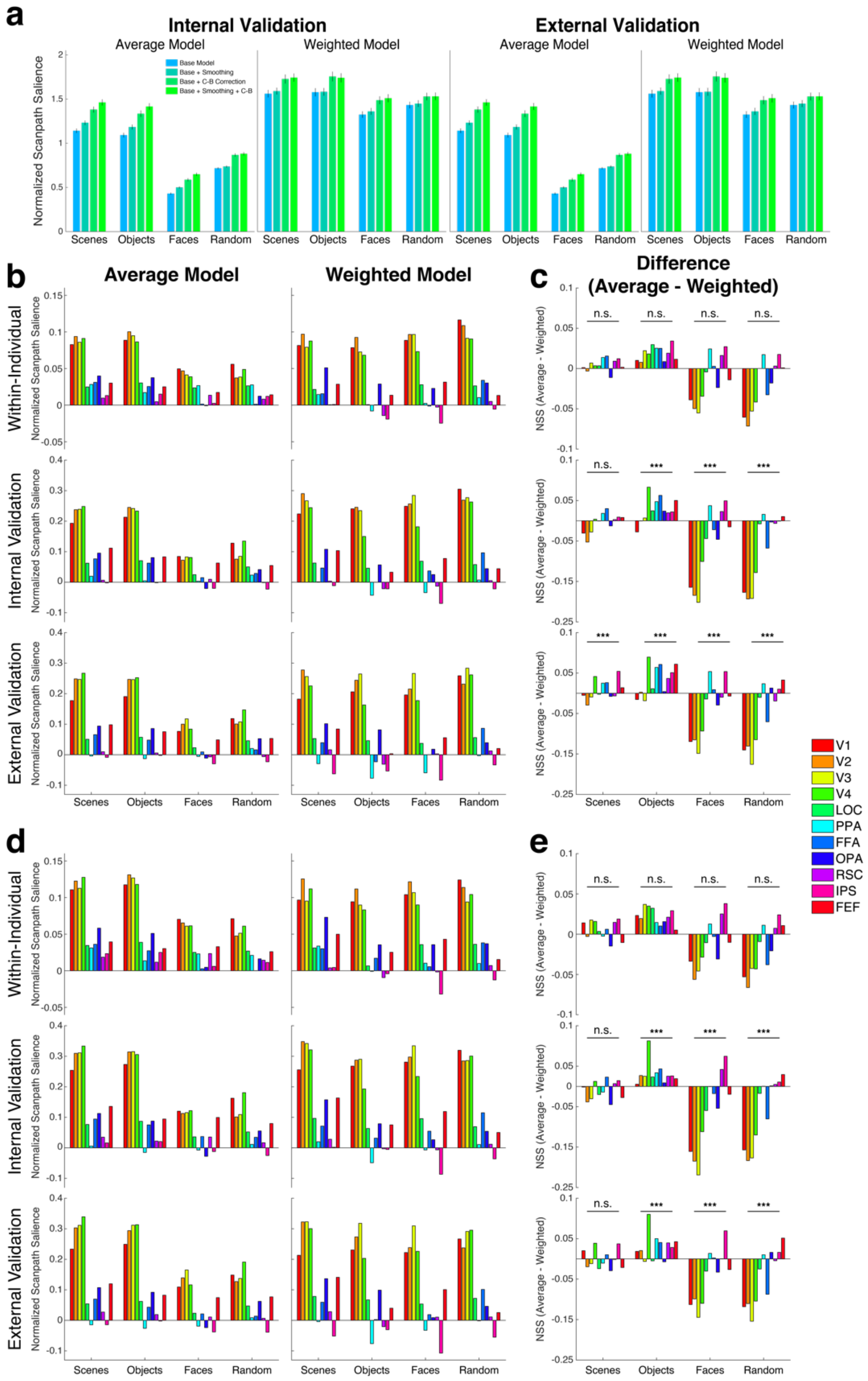

Supplementary Fig. 1. Prediction results for models that average across the CNN channel dimension relative to models that take a weighted sum across the CNN channel dimension. (**a**) Prediction performance for computational spatial attention models. Error bars represent standard error of the mean across participants in the internal (*n = 11*) and external (*n = 22*) validation sets. (**b**) Prediction performance for base model-based reconstructions. *NSS* scores are significant in V1-hV4 for all analyses. (**c**) Difference *NSS* scores between average and weighted models for base reconstructions. Markers indicate significance for a main effect of model type (average vs weighted) in a 2-way repeated-measures ANOVA with model type and ROI as factors. (**d**) Prediction performance for smoothed and center-bias corrected model-based reconstructions. *NSS* scores were significant in V1-hV4 for all analyses. (**e**) Difference *NSS* scores between average and weighted models for smoothed and center-bias corrected reconstructions. ***$p<0.001$, main effect of model type (average vs weighted).

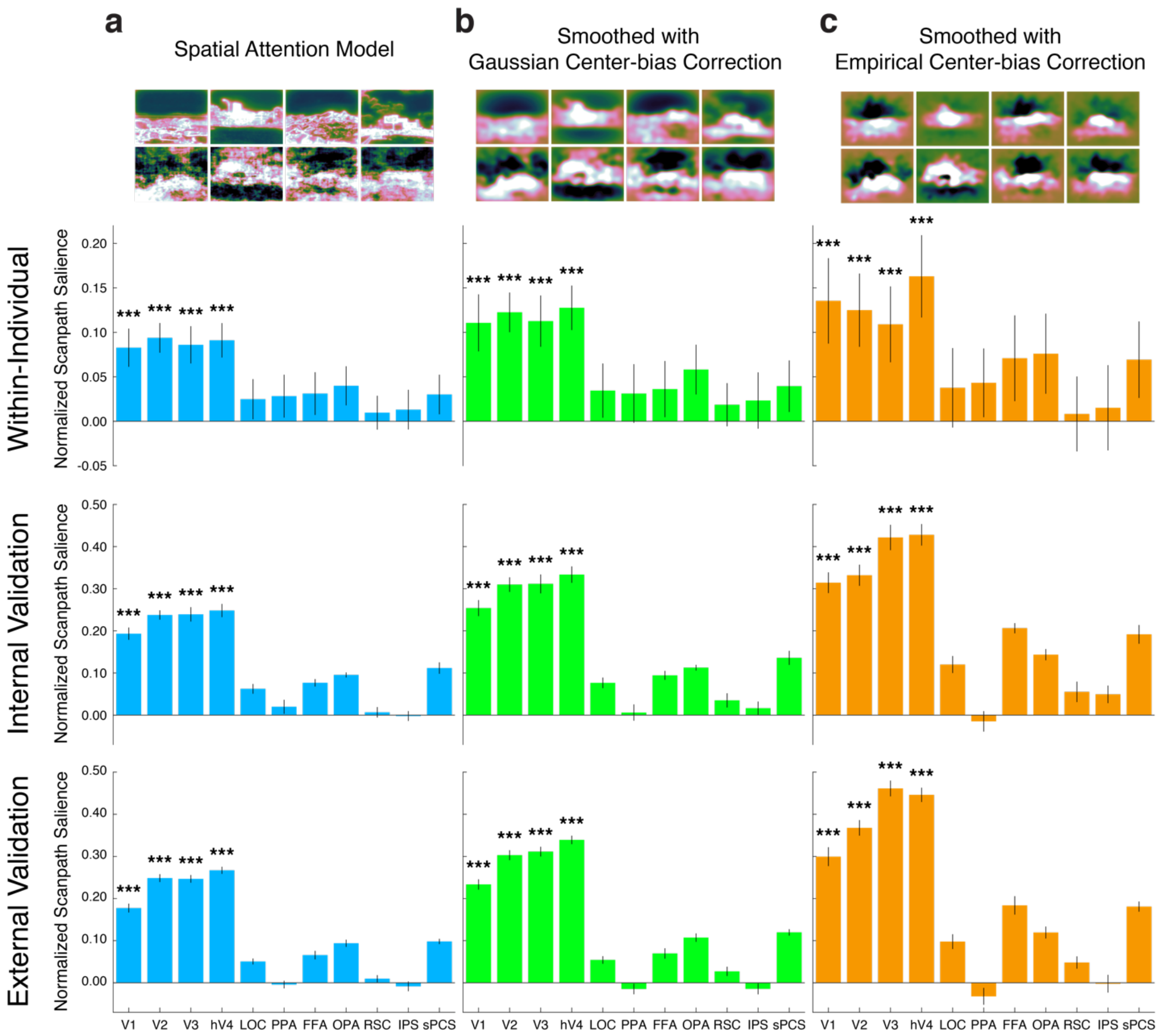

Supplementary Fig. 2. Empirical center-bias correction (**c**) improves prediction performance. Results for base reconstructions (**a**) and reconstructions center-bias corrected with a 2D Gaussian (**b**) are included for comparison. Error bars represent standard error of the mean across participants in the fMRI dataset (*n = 11*, Within-Individual and Internal Validation) and external validation dataset (*n = 22*, External Validation). Significance is defined using permutation testing. * $P < 1 \times 10^{-2}$, ** $P < 4.55 \times 10^{-3}$ (Bonferroni-corrected threshold), *** $P < 1 \times 10^{-3}$.

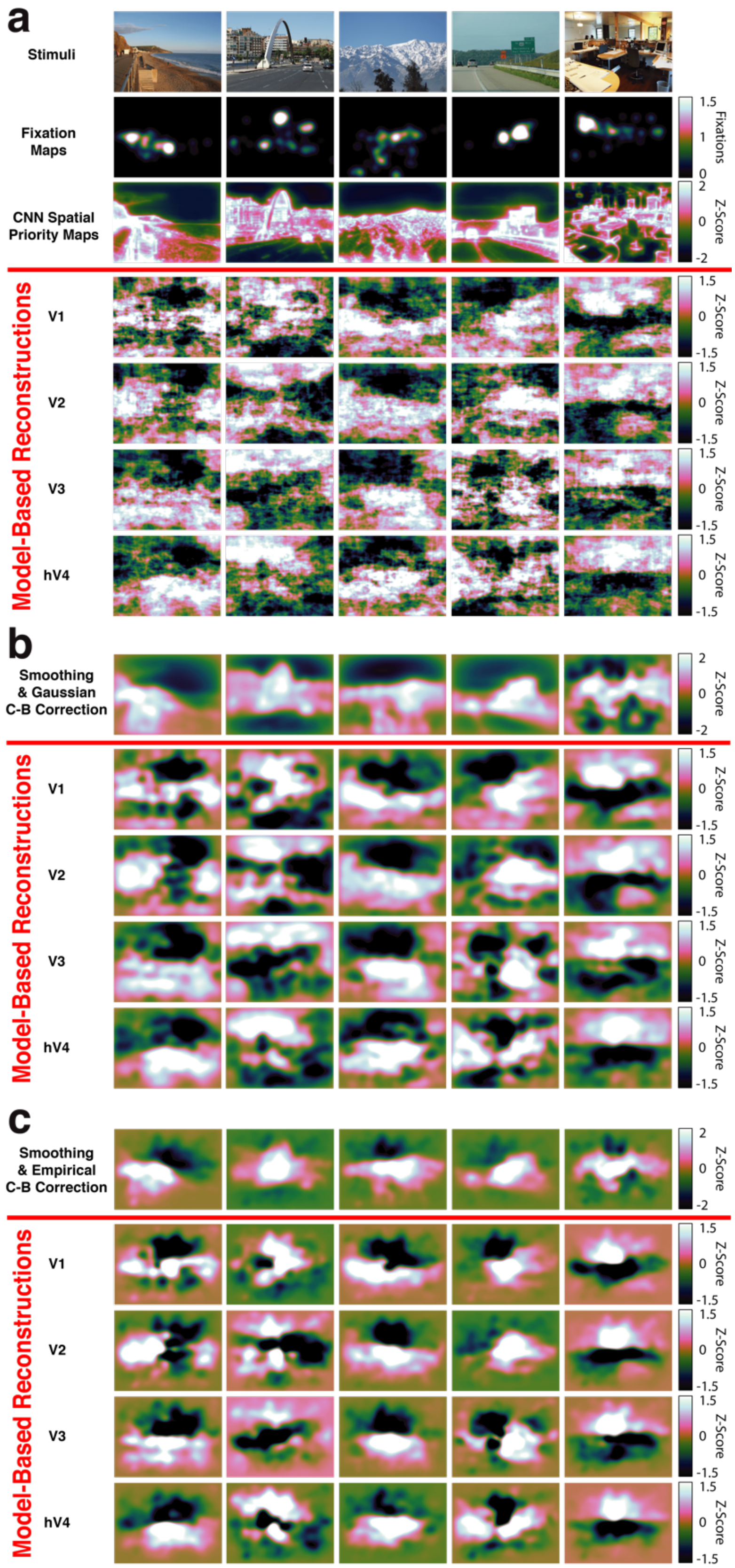

Supplementary Fig. 3. Model-based reconstructions empirically corrected for center-bias (**c**). Base reconstructions (**a**) and reconstructions corrected for center-bias with a 2D Gaussian (**b**) are included for comparison.

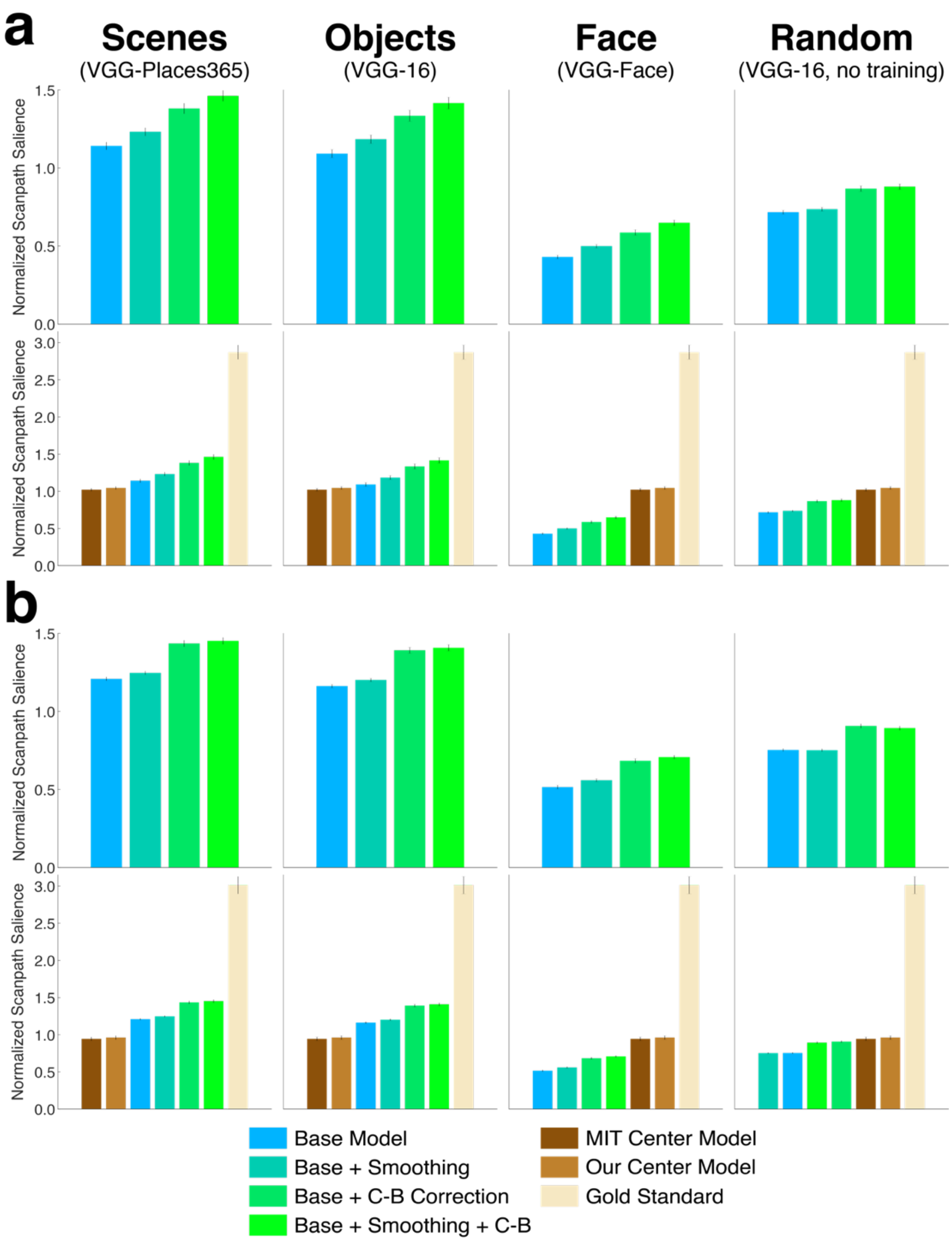

Supplementary Fig. 4. Computational spatial attention model results for all CNN types, and results for all CNN types and benchmark measures sorted by prediction accuracy. **a.** Internal validation. **b.** External validation. Error bars represent standard error of the mean across all participants in the internal (*n* = *11*) and external (*n* = *22*) validation sets).

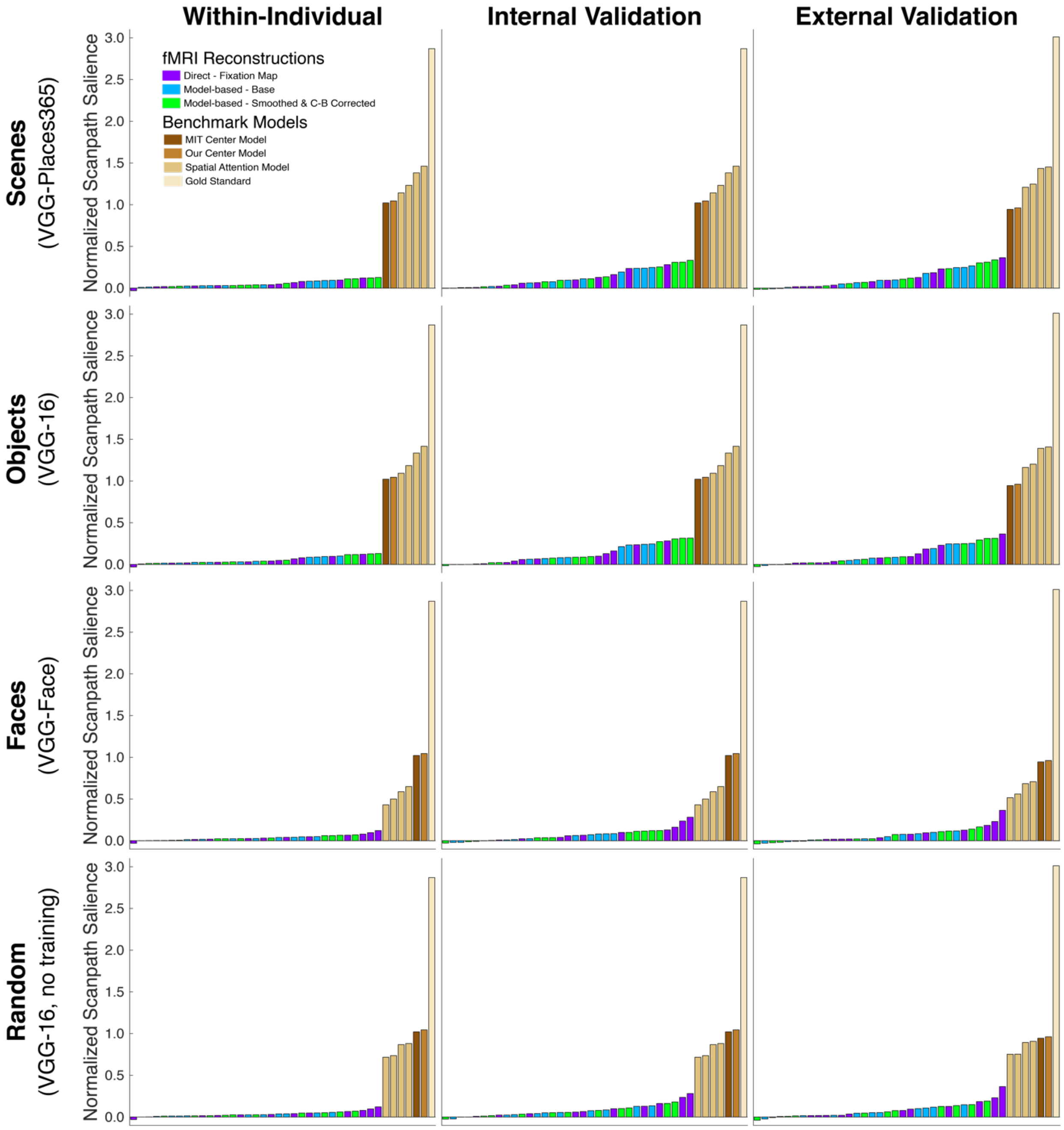

Supplementary Fig 5. Prediction performance for fMRI reconstruction, computational spatial attention models, and benchmark models. Results are sorted by performance predicting eye movement patterns.

SUPPLEMENTARY REFERENCES

1. Judd, T., Ehinger, K. A., Durand, F. & Torralba, A. Learning to Predict Where Humans Look. in 1–8 (2009).
